# Complex of GM1- and GD1a-Like Lipo-Oligosaccharide Mimics GM1b, Inducing Anti-GM1b Antibodies

**DOI:** 10.1371/journal.pone.0124004

**Published:** 2015-04-13

**Authors:** Michiaki Koga, Michel Gilbert, Jianjun Li, Nobuhiro Yuki

**Affiliations:** 1 Department of Neurology, Dokkyo Medical University, Tochigi, Japan; 2 Department of Neurology and Clinical Neuroscience, Yamaguchi University Graduate School of Medicine, Yamaguchi, Japan; 3 Human Health Therapeutics, National Research Council Canada, Ottawa, Ontario, Canada; 4 Departments of Medicine and Physiology, National University of Singapore, Singapore, Singapore; Medical University of Innsbruck, AUSTRIA

## Abstract

**Objective:**

Molecular mimicry between *Campylobacter jejuni* lipo-oligosaccharides (LOSs) and human gangliosides GM1 and GD1a induces the production of anti-GM1 and anti-GD1a antibodies, and the development of Guillain-Barré syndrome. Complexes of two different gangliosides form new molecular shapes capable of enhancing recognition by anti-ganglioside antibodies. To test the hypothesis that the complex of GM1-like and GD1a-like LOSs of *C*. *jejuni* induces the development of anti-GM1b antibodies in Guillain-Barré syndrome patients.

**Methods:**

Mass spectrometry analysis determined the LOS outer core structures, with which mice were immunized. IgG antibodies to single gangliosides and complex of gangliosides were tested in sera from Guillain-Barré syndrome patients from whom *C*. *jejuni* LOS had been isolated.

**Results:**

Two isolates from GBS patients who had anti-GM1b antibodies, but neither anti-GM1 nor -GD1a antibodies, expressed both GM1-like and GD1a-like LOSs, but not GM1b-like LOS. Anti-GM1b antibodies were induced in one of the mice immunized with the *C*. *jejuni* bearing GM1-like and GD1a-like LOS. Sera from 20 patients had antibodies to the complex of GM1 and GD1a, all of which carried anti-GM1b reactivity. Five of these sera harbored neither anti-GM1 nor anti-GD1a antibodies. IgG antibodies to the complex were absorbed by GM1b, but by neither GM1 nor GD1a.

**Conclusions:**

GM1-like and GD1a-like LOSs form a GM1b epitope, inducing the development of anti-GM1b antibodies in patients with Guillain-Barré syndrome subsequent to *C*. *jejuni* enteritis. Here, we present a new paradigm that the complex of two different structures forms a new molecular mimicry, inducing the production of autoantibodies.

## Introduction

Molecular mimicry between *Campylobacter jejuni* lipo-oligosaccharides (LOSs) and human gangliosides GM1 and GD1a induces the production of anti-GM1 and anti-GD1a IgG antibodies, and the development of axonal Guillain-Barré syndrome (GBS) [1, 2]. GM1b is a component of human peripheral nerves, and anti-GM1b IgG antibodies are also associated with axonal GBS, subsequent to *C*. *jejuni* enteritis [3, 4]. Some patients with GBS have no antibodies to single gangliosides, but have antibodies to heteromeric complexes of two different gangliosides when mixed in 1:1 molar ratio [5]. Heteromeric complexes are defined as structurally distinct gangliosides that interact to form new molecular shapes capable of enhancing recognition by anti-ganglioside antibodies [6]. A combinatorial glycoarray methodology was recently used to assess the frequency of glycolipid complex antibodies in a cohort of GBS patients [7]. The inclusion of glycolipid complexes increased the positivity rate of the sera from patients with the demyelinating form of GBS and antibodies against specific complexes were found to be associated with particular clinical features.[1]Infection by *C*. *jejuni* bearing two different ganglioside-like LOSs may induce the production of antibodies against ganglioside complexes [8].

**Fig 1:**
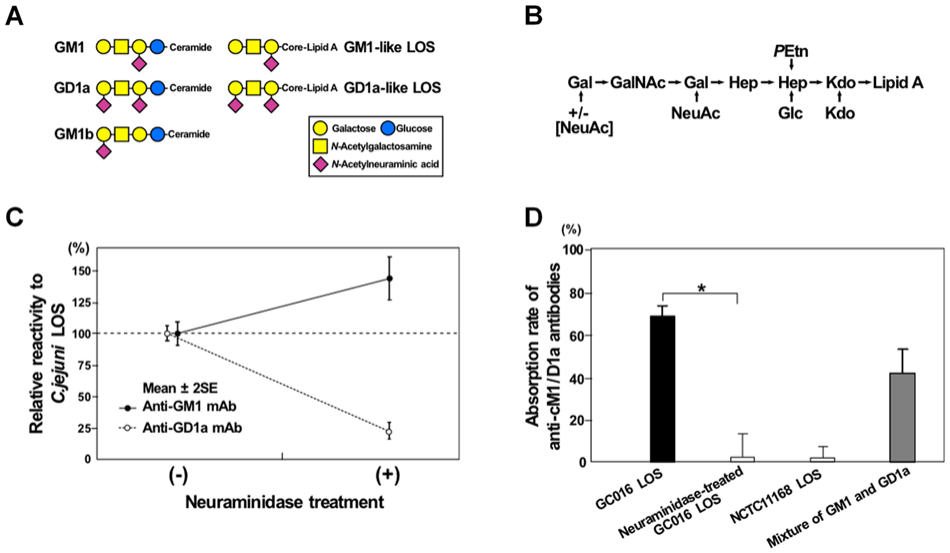
GM1-like and GD1a-like lipo-oligosccharides (LOSs). (A) Schematic structures of GM1, GD1a and GM1b gangliosides, as well as GM1-like and GD1a-like LOSs of *Campylobacter jejuni*. (B) Proposed LOS outer core structure of *C*. *jejuni* strains (GC016 and GC105) isolated from patients with GBS who had anti-GM1b antibodies, but neither anti-GM1 nor anti-GD1a antibodies. Gal = Galactose; NeuAc = *N*-Acetylneuraminic acid; GalNAc = *N*-Acetylgalactosamine; Hep = L-*glycero*-D-*manno*-Heptose; Glc = Glucose; Kdo = 3-deoxy-D-*manno*-2-Octulosonic acid; *P*Etn = Phosphoethanolamine. (C) *C*. *jejuni* (GC016) LOS with and without neuraminidase treatment. Anti-GD1a monoclonal antibody reactivity to the LOS was decreased after the treatment, whereas anti-GM1 monoclonal antibody reactivity was increased. This indicates that the neuraminidase-treatment transformed GD1a-like LOS into GM1-like LOS by removing the terminal neuraminic acid. (D) Absorption studies using antibodies against cM1/D1a with various antigens. Anti-cM1/D1a IgG antibodies from the serum of a patient with GBS (S382) were absorbed by the intact GC016 LOS (black bar; GM1/GD1a mimics) and a mixture of GM1 and GD1a gangliosides (gray bar), but not by neuraminidase-treated GC016 LOS (GM1 mimic as shown in A) and NCTC11168 LOS (GM1/GM2 mimics).

To identify the mechanism by which the anti-GM1b antibodies are induced, we analyzed the LOS outer core structure of *C*. *jejuni* strains isolated from GBS patients who had anti-GM1b antibodies. Unexpectedly, however, we found that the isolates expressed GM1 and GD1a mimics, but not GM1b mimic (Fig 1A). In the current study, we tested a working hypothesis that a complex of GM1-like and GD1a-like LOSs forms a new epitope, inducing the development of anti-GM1b antibodies.

## Methods

### Serum samples and *C*. *jejuni* strains

Sera were available from 119 of 138 patients with *C*. *jejuni*-isolated GBS and related conditions at the acute phase [9]. Written informed consent was obtained from all the patients, and the Ethical Committee of Dokkyo Medical University, Japan, approved the performance of this study. LOS biosynthesis locus and *cstII* genotype (Thr/Asn51) were determined by PCR screening of specific genes and by sequencing of the *cstII* gene as previously described [9, 10].

### Mass spectrometry analysis

*C*. *jejuni* was grown overnight on a single agar plate and the cells were treated with proteinase K, RNAse A and DNAse I as previously described [10]. The digested cells were treated with hydrazine to cleave *O*-linked fatty acids and the *O*-deacylated LOS samples were analyzed by capillary electrophoresis coupled to electrospray ionization mass spectrometry [11].

### Mice immunization

Mice lacking the functional gene for (*N*-acetylneuraminyl)-galactosylglucosylceramide *N*-acetylgalactosaminyltransferase are immune naive hosts against gangliosides, and show a strong IgG response to GM1-like and GD1a-like LOSs [12]. A *C*. *jejuni* strain (GC105) isolated from a patient with GBS carries both GM1-like and GD1a-like LOSs as described below, whereas genome strain NCTC11168 bears GM1-like and GM2-like LOSs, but no GD1a-like LOS [13]. The mice were immunized intraperitoneally 5 times at 2-week intervals with 1 mg (dry weight) of heat-killed lysate of *C*. *jejuni* [14]. This research was approved by the Animal Care and Use Committee, Dokkyo Medical University, Japan (approval no. 00–22). The mice were treated according to the Guidelines for the Care and Use of Laboratory Animals, Dokkyo Medical University, Japan.

### Enzyme-linked immunosorbent assay

IgG antibodies to individual gangliosides (GM1, GM1b, GM2, GD1a, GalNAc-GD1a, GD1b, GD2, GT1a, GT1b or GQ1b; 10 pmol/well) were measured in sera (starting at 1:500 dilution) from the patients and mice using peroxidase-conjugated anti-human or anti-mouse IgG antibodies [15]. IgG antibodies to ganglioside complex GM1/GD1a (cM1/D1a) were tested with a mixture of GM1 and GD1a (each 5 pmol/well) as antigen. Anti-cM1/D1a antibodies were judged positive when the optical density of the antibodies was 0.5 greater than the sum of optical densities of antibodies to individual GM1 and GD1a [16]. IgG antibodies to other ganglioside complexes were measured as well. Frequency differences between the groups were compared by means of Fisher’s exact test using SPSS 12.0J software (SPSS Inc., Chicago, IL). A difference was considered significant when the two-sided *p* value was less than 0.05. Absorption studies were performed as described elsewhere [17]. Absorption rates (%) were calculated from [1−(optical densities in wells with serum with absorption-treatment) / (optical densities in reference wells with serum without absorption-treatment)] x 100.

Crude LOS fractions were prepared from *C*. *jejuni* strains, and the presence of GM1-like, GD1a-like, GM1/GD1a-like or GM1b-like LOS was determined using monoclonal antibodies [anti-GM1 (GB2) and anti-GD1a (GB1)], cM1/D1a serum (S382) or GM1b-specific serum (S8056) from patients with GBS as described elsewhere [18]. GD1a-like LOS was treated overnight with the neuraminidase from *Arthrobacter ureafaciens* (Nakalai Tesque, Inc., Kyoto, Japan) at a concentration of 0.3 mU/ml acetate buffer (pH 5.0) at 37°C. This treatment in the absence of surfactant is expected to cleave only the terminal sialic acid in the ganglioside.

### Thin-layer chromatography with immunostaining

Bovine brain ganglioside mixture, authentic GM1b and a mixture of GM1 and GD1a were spotted on a thin-layer chromatography plate and developed with chloroform/methanol/12 mM magnesium chloride in water (5:4:1, by volume) [19]. After the development, a mixture of GM1 and GD1a was spotted on a different lane. Each plate was overlaid with serum (1:50 dilution,) from *Patient 4* in Table 1, followed by peroxidase-conjugated anti-human IgG antibodies.

**Table 1:** IgG antibody titers in Guillain-Barré syndrome patients from whom *C*. *jejuni* was isolated and who had anti-GM1/GD1a complex antibodies, but neither anti-GM1 nor anti-GD1a antibodies.

		IgG antibodies to (titer)	
Patient	Age/Sex	Isolated ganglioside ^a^	Ganglioside- complex ^b^
1	24/M	GM1b (32,000)	GM1/GD1a (16,000)
			GM1/GT1b (4,000)
			GD1a/GD1b (500)
2	81/F	GM1b (16,000)	GM1/GD1a (500)
3	26/M	GM1b (32,000)	GM1/GD1a (1,000)
4	16/F	GM1b (128,000)	GM1/GD1a (8,000)
			GM1/GT1b (4,000)
			GD1a/GD1b (1,000)
5	13/M	GM1b (64,000)	GM1/GD1a (2,000)
		GD1b (2,000)	GM1/GT1b (1,000)
			GD1a/GD1b (1,000)

^a^Tested antigens were GM1, GM1b, GM2, GD1a, GalNAc-GD1a, GD1b, GD2, GT1a, GT1b, and GQ1b gangliosides.

^b^Tested antigens were GM1/GD1a, GM1/GD1b, GM1/GT1b, GD1a/GD1b, GD1a/GT1b, and GD1b/GT1b complexes.

## Results

### Structural characterization of *C*. *jejuni* LOSs from patients with GBS associated with anti-GM1b antibodies

*Patients 1* to *5* with GBS harbored the anti-GM1b antibodies, but neither anti-GM1 nor anti-GD1a antibodies (Table 1). All the 5 patients had anti-cM1/D1a IgG antibodies. Monoclonal antibodies detected both GM1 and GD1a epitopes on the LOSs of the *C*. *jejuni* isolates obtained from the 5 patients. All the 5 isolates had class A LOS biosynthesis locus and *cstII* encoding the mono-functional CstII (Thr51) variant, both of which are associated with the biosynthesis of GM1-like and GD1a-like LOSs [20]. Although these genotypic results suggested GM1 and GD1a mimicry, structural analysis was required to rule out the presence of a GM1b epitope that could have triggered the anti-GM1b antibody production in these patients.

Capillary electrophoresis coupled to electrospray ionization mass spectrometry (CE-ESI-MS) was performed to analyze *O*-deacylated samples and to propose the LOS outer core structures for the 2 isolates (GC016 and GC105) from *Patients 1* and *3*, respectively. The CE-ESI-MS data showed mass species with either 1 or 2 sialic acids (S1 Table), which are proposed to be derived from GM1 and GD1a mimicry (Fig 1B). The glycosyltransferase variants in the LOS biosynthesis locus of GC016 and GC105 are consistent with GM1/GD1a mimicry with a mono-functional CstII (Thr51) that will transfer a single sialic acid to the galactose residues. GM1b mimicry was ruled out because the CgtA (β-1,4-*N*-acetylgalactosaminyltransferase) and CgtB (β-1,3-galactosyltransferase) variants are specific to sialylated acceptors, which implies that the inner galactose residue must be substituted with a sialic acid. Based on the relative abundance of the quadruply charged ions for each mass species (data not shown), we estimated that the ratio of GM1:GD1a mimics was 1:3 for GC016, whereas it was estimated to be 3:1 for GC105.

GM1-like and GD1a-like LOSs of *C*. *jejuni* (GC016) were treated with the *A*. *ureafaciens* neuraminidase. The treatment resulted in a decrease of the anti-GD1a antibody binding to the LOS and an increase of the anti-GM1 antibody binding (Fig 1C), indicating that GD1a-like LOS was converted to GM1-like LOS. cM1/D1a serum (S382) IgG antibodies were absorbed by cM1/D1a and GM1/GD1a-like LOS, but not by the neuraminidase-treated GC016 LOS or GM1/GM2-like LOS (Fig 1D). In contrast, the GM1b-specific serum (S8056) bound to the GM1/GD1a-like LOS, but not to the GM1-like LOS alone, when tested by enzyme-linked immunosorbent assay and thin-layer chromatography-immunostaining (data not shown). These data suggest that complex formation of GM1-like and GD1a-like LOSs could induce the production of anti-GM1b antibodies in such patients.

### Induction of anti-GM1b antibodies in mice

Sensitization of immune-naive mice with *C*. *jejuni* (GC105) bearing GM1-like and GD1a-like LOSs induced the production of high titers (OD >1.0) of anti-GM1b antibodies in 4 of 13 mice, as well as anti-GM1 antibodies in 10 mice and anti-GD1a antibodies in 9 mice. In contrast, immunization with *C*. *jejuni* (NCTC11168) bearing GM1-like and GM2-like LOSs did not induce the production of anti-GM1b antibodies in any of the 8 mice tested, and resulted in the induction of anti-GM1 antibodies in 7 mice and anti-GD1a antibodies in 5 mice.

### Cross-reactive antibodies to cM1/D1a with GM1b

**Fig 2:**
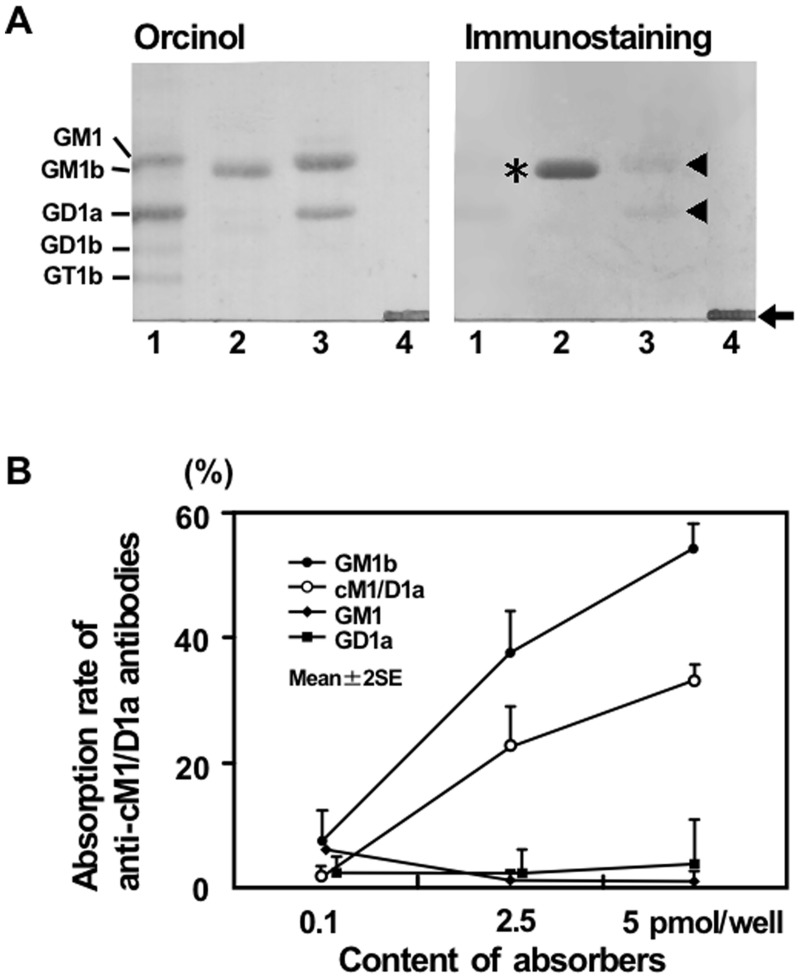
Complex of GM1 and GD1a (cM1/GD1a). (A) Immunostaining with IgG antibodies from *Patient 4*. Bovine brain ganglioside mixture (lane 1), authentic GM1b (lane 2) and a mixture of GM1 and GD1a (lane 3) were spotted on a thin-layer chromatogram plate and developed with a solvent. After the development of the plate, a mixture of GM1 and GD1a was spotted and not further resolved (lane 4). The patient’s IgG strongly bound to GM1b (asterisk) with the development, and to the mixture of GM1 and GD1a that was not separated (arrow). In contrast, the binding nearly disappeared after separation of GM1 and GD1a with the development (arrow head). (B) Cross-reactivity of the antibodies to cM1/D1a with GM1b. IgG anti-cM1/D1a antibodies of serum (S6325) from a patient with GBS subsequent to *C*. *jejuni* enteritis were dose-dependently absorbed by GM1b, as well as by cM1/D1a, whereas the antibodies were absorbed by neither GM1 nor GD1a.

Anti-cM1/D1a IgG antibodies were found in 20 of the 119 patients with neuropathy and its related conditions from whom *C*. *jejuni* had been isolated, whereas no anti-cM1/D1a antibodies were detected in the sera from 105 healthy subjects and 83 patients with other neurological disorders such as amyotrophic lateral sclerosis and myasthenia gravis. Five of the 20 patients carried anti-cM1/D1a IgG antibodies (1:500 to 1:16,000), but neither anti-GM1 nor anti-GD1a IgG antibodies (Table 1). All the 5 patients had GBS, but not Miller Fisher syndrome. Surprisingly, all of the 5 patients carrying anti-cM1/D1a antibodies had high titers of anti-GM1b IgG antibodies (1:16,000 to 1:128,000). Fig 2A demonstrates that IgG antibodies from *Patient 4* in Table 1 reacted with GM1b and cM1/D1a, but with neither GM1 nor GD1a.

Anti-cM1/D1a IgG antibodies from the patients were dose-dependently absorbed by GM1b, as well as by cM1/D1a, but by neither GM1 nor GD1a, indicating that anti-cM1/D1a antibodies cross-react with GM1b. Fig 2B shows representative results using serum from a patient with GBS after *C*. *jejuni* enteritis (S6325). Anti-GM1b antibodies also were absorbed by cM1/D1a, as well as by GM1b, but by neither GM1 nor GD1a.

### Anti-GM1b antibodies and *C*. *jejuni* LOSs

Based on the ganglioside-mimicking pattern of *C*. *jejuni* LOSs, the 119 neuropathic patients were classified into three groups; (i) both GM1 and GD1a mimics, (ii) GM1 mimic alone, and (iii) neither GM1 nor GD1a mimic (Table 2). The first group of patients more often had IgG antibodies to GM1 and GD1a, as well as to cM1/D1a, than the others, and each difference was statistically significant. The autoantibody response was dependent on individuals: For example, some patients infected by *C*. *jejuni* bearing GM1 and GD1a epitopes showed specific anti-GM1 antibodies elevation (35%), and others carried both anti-GM1 and -GD1a antibodies, with no anti-cM1/D1a activity (28%). Expectedly, the neuropathic patients infected by *C*. *jejuni* carrying GM1 and GD1a epitopes had IgG autoantibodies to GM1b more frequently than the others.

**Table 2:** IgG antibodies to GM1, GD1a, GM1/GD1a complex and GM1b in neuropathic patients from whom *Campylobacter jejuni* was isolated.

		IgG antibodies to			
Ganglioside mimics of *C*. *jejuni* isolates				GM1/GD1a	
	*n*	GM1	GD1a	complex	GM1b
Both GM1 and GD1a	57	42 (74%)^a^	25 (44%)^b^	14 (25%)^c^	36 (63%)^d^
Only GM1	11	7 (64%)	4 (36%)	1 (9%)	6 (55%)
Neither GM1 nor GD1a	51	11 (22%)	8 (16%)	5 (10%)	13 (25%)

Patients are subgrouped based on ganglioside mimics on the lipo-oligosaccharide of their *C*. *jejuni* isolate. Significant increase compared to other patients

^a^ *p* <0.001, odds ratio [OR] = 6.8, 95% confidence interval [CI] = 3.1–15.3;

^b^ *p* = 0.005, OR = 3.3, 95% CI = 1.4–7.4;

^c^ *p* = 0.048, OR = 3.0, 95% CI = 1.1–8.6;

^d^ *p* <0.001, OR = 3.9, 95% CI = 1.8–8.3.

## Discussion

In the current study, *C*. *jejuni* strains expressing GM1 and GD1a mimics, but not GM1b mimics, were isolated from GBS patients who harbored anti-GM1b antibodies. More importantly, one of the strains (GC105) could induce the development of anti-GM1b antibodies in mice. In a comprehensive study, 5 of 26 *C*. *jejuni* strains from GBS and Miller Fisher syndrome expressed GM1b mimics [21]. Anti-ganglioside antibody results were not available in that study, but the presence of a GM1b mimic in a *C*. *jejuni* strain would provide a straightforward mechanism to explain an anti-GM1b antibody response. In the same study, 8 of the strains isolated from GBS patients expressed GM1 and GD1a mimics. Since two authors (J.L. and M.G.) were involved in the structural characterization in both studies, the same analytical methods were applied to make sure we did not overlook the possible presence of GM1b mimics in the strains from the current study.

All the GBS patients who carried the anti-cM1/D1a antibodies harbored anti-GM1b antibodies, and 5 of them had neither anti-GM1 nor anti-GD1a antibodies. The anti-cM1/D1a antibodies were absorbed by GM1b, and the anti-GM1b antibodies were absorbed by cM1/D1a, suggesting that cM1/D1a forms a GM1b epitope. This was supported by the induction of the anti-GM1b antibodies in mice immunized with a *C*. *jejuni* strain (GC105) bearing both GM1-like and GD1a-like LOSs. Two-thirds of the patients with neuropathy from whom *C*. *jejuni* strains bearing GM1 and GD1a epitopes were isolated had the anti-GM1b antibodies (Table 2). In one study, a *C*. *jejuni* strain was shown to have LOS mimicking GM1 and GD1a, and the patient with GBS from whom the strain was isolated had higher titer (1:12,800) of anti-cM1/D1a IgG antibodies, lower titer (1:800) of anti-GM1 IgG antibodies and no anti-GD1a antibodies [8]. The anti-GM1b antibody result was not available, but this patient might have had anti-GM1b antibodies, based on the high titer of anti-cM1/D1a antibodies.

In the current study, immunization with *C*. *jejuni* bearing GM1-like and GD1a-like LOSs induced the development of anti-GM1b antibodies in some mice, and anti-GM1 or anti-GD1a antibodies in others. In an earlier study, similarly, some patients with GBS carried anti-GM1b and anti-GM1 antibodies, whereas others carried either only anti-GM1 or anti-GM1b antibodies [22].

In conclusion, GM1-like and GD1a-like LOSs may form a GM1b epitope, inducing the development of anti-GM1b antibodies. The exact structural basis for the presentation of a GM1b epitope does not seem to rely on the relative proportions of GM1-like and GD1a-like in the LOS, since we observed very different ratios of GM1:GD1a mimics (3:1 vs 1:3) in the two strains that were analyzed by mass spectrometry. In this study, we have presented a new paradigm, demonstrating that the complex of two different structures form a new molecular mimicry, inducing the production of autoantibodies.

GM1 and GD1a are strongly expressed in the human peripheral nerves, whereas GM1b is weakly expressed in these tissues [3]. GM1 and GD1a form a heteromeric complex in murine peripheral nerves [23]. Along with our findings, both GM1b and cM1/D1a may be targets of anti-GM1b and anti-cM1/D1a antibodies in the peripheral nerves. Infection by *C*. *jejuni* bearing GM1 and GD1a epitopes may induce the production of anti-GM1b antibodies, which bind to GM1b itself or to a heteromeric complex of GM1 and GD1a at the nodes of Ranvier and activate complement in the peripheral motor nerves. As shown in a rabbit model of axonal GBS [24], the autoimmune attack should result in the disappearance of voltage-gated sodium channel clusters and disruption of the paranodal junctions, leading to motor nerve conduction failure and muscle weakness in patients with GBS.

## Supporting Information

S1 Table Negative ion electrospray ionization mass spectrometry data and proposed compositions for *O*-deacylated LOS of *C*. *jejuni* GC016 and GC105. (DOC)
