# Supplementary material for: Complex of GM1- and GD1a-Like Lipo-Oligosaccharide Mimics GM1b, Inducing Anti-GM1b Antibodies
Source: PLoS One. 2015 Apr 13;10(4):e0124004. doi: 10.1371/journal.pone.0124004 (PMC4395030; doi:10.1371/journal.pone.0124004)
Supplement: S1 Table — (DOC) [file pone.0124004.s001.doc]

**Supplementary Table S1.** Negative ion electrospray ionization mass spectrometry data and proposed compositions for *O*-deacylated LOS of *C. jejuni* GC016 and GC105.

| **Strain** | **Observed ions**  **(*m/z*)** | | | **Molecular mass**  **(Da)** | | **Proposed compositions** | | |
| --- | --- | --- | --- | --- | --- | --- | --- | --- |
|  | **[M‑5H]^5-^** | **[M‑4H]^4-^** | **[M-3H]^3-^** | **Observed** | **Calculated[[1]](#endnote-2)** | **Core oligosaccharide** | **Phosphorylation in lipid A** | **Acylation in lipid A** |
| GC016 | 644.8 | 806.5 | 1075.6 | 3229.6 | 3230.07 | Neu5Ac_1_•HexNAc_1_•Hex_3_•Hep_2_•*P*Etn_1_•Kdo_2_ | *PP*Etn, *P* | 3 *N*-(C14:0 3-OH) |
|  | 669.5 | 837.2 | 1116.5 | 3352.6 | 3353.12 | Neu5Ac_1_•HexNAc_1_•Hex_3_•Hep_2_•*P*Etn_1_•Kdo_2_ | *PP*Etn, *PP*Etn | 3 *N*-(C14:0 3-OH) |
|  | 703.4 | 879.2 | 1172.8 | 3521.4 | 3521.33 | Neu5Ac_2_•HexNAc_1_•Hex_3_•Hep_2_•*P*Etn_1_•Kdo_2_ | *PP*Etn, *P* | 3 *N*-(C14:0 3-OH) |
|  | 728.0 | 910.1 | 1213.8 | 3644.6 | 3644.38 | Neu5Ac_2_•HexNAc_1_•Hex_3_•Hep_2_•*P*Etn_1_•Kdo_2_ | *PP*Etn, *PP*Etn | 3 *N*-(C14:0 3-OH) |
|  | 748.2 | 935.1 | 1246.6 | 3744.4 | 3746.71 | Neu5Ac_2_•HexNAc_1_•Hex_3_•Hep_2_•*P*Etn_1_•Kdo_2_ | *PP*Etn, *P* | 4 *N*-(C14:0 3-OH) |
|  | 773.1 | 966.1 |  | 3869.5 | 3869.76 | Neu5Ac_2_•HexNAc_1_•Hex_3_•Hep_2_•*P*Etn_1_•Kdo_2_ | *PP*Etn, *PP*Etn | 4 *N*-(C14:0 3-OH) |
| GC105 | 645.1 | 806.4 | 1075.5 | 3229.9 | 3230.07 | Neu5Ac_1_•HexNAc_1_•Hex_3_•Hep_2_•*P*Etn_1_•Kdo_2_ | *PP*Etn, *P* | 3 *N*-(C14:0 3-OH) |
|  | 669.7 | 837.3 | 1116.7 | 3353.3 | 3353.12 | Neu5Ac_1_•HexNAc_1_•Hex_3_•Hep_2_•*P*Etn_1_•Kdo_2_ | *PP*Etn, *PP*Etn | 3 *N*-(C14:0 3-OH) |
|  | 690.2 | 863.1 | 1150.5 | 3455.6 | 3455.45 | Neu5Ac_1_•HexNAc_1_•Hex_3_•Hep_2_•*P*Etn_1_•Kdo_2_ | *PP*Etn, *P* | 4 *N*-(C14:0 3-OH) |
|  | 703.4 | 879.3 | 1172.8 | 3521.5 | 3521.33 | Neu5Ac_2_•HexNAc_1_•Hex_3_•Hep_2_•*P*Etn_1_•Kdo_2_ | *PP*Etn, *P* | 3 *N*-(C14:0 3-OH) |
|  | 714.8 | 893.9 | 1192.1 | 3579.3 | 3578.50 | Neu5Ac_1_•HexNAc_1_•Hex_3_•Hep_2_•*P*Etn_1_•Kdo_2_ | *PP*Etn, *PP*Etn | 4 *N*-(C14:0 3-OH) |
|  | 748.3 | 936.0 | 1248.1 | 3747.3 | 3746.71 | Neu5Ac_2_•HexNAc_1_•Hex_3_•Hep_2_•*P*Etn_1_•Kdo_2_ | *PP*Etn, *P* | 4 *N*-(C14:0 3-OH) |

1. Isotope-average mass units were used for calculation of molecular mass values based on proposed compositions as follows: Hex,162.14; HexNAc, 203.20; Hep, 192.17; Kdo, 220.18; *P*, 79.98; *P*Etn, 123.05; Neu5Ac, 291.26; HexN, 161.16; HexN3N, 160.18; C14:0 3-OH, 226.36; H_2_O, 18.01. [↑](#endnote-ref-2)
